# The Economic Burden of Otitis Media in Korea, 2012: A Nationally Representative Cross-Sectional Study

**DOI:** 10.1155/2016/3596261

**Published:** 2016-09-14

**Authors:** Young-Eun Kim, Ye-Rin Lee, So-Youn Park, Kyung Suk Lee, In-Hwan Oh

**Affiliations:** ^1^Department of Preventive Medicine, School of Medicine, Kyung Hee University, Seoul, Republic of Korea; ^2^Department of Medical Education and Humanities, School of Medicine, Kyung Hee University, Seoul, Republic of Korea; ^3^Department of Pediatrics, CHA Bundang Medical Center, CHA University School of Medicine, Gyeonggi-do, Republic of Korea

## Abstract

*Background*. Otitis media (OM) is a common communicable disease that is associated with a substantial economic burden. However, no Korean studies have evaluated OM-related trends after the introduction of the pneumococcal conjugate vaccines.* Purpose*. This study aimed to estimate the prevalence and economic burden of OM in Korea using nationally representative data.* Methods*. The burden of OM was estimated nationally representative data such as national health insurance claims data from 2012, based on the prevalence approach and the societal perspective.* Results*. In 2012, 1,788,303 patients visited medical institutions for treatment of OM, and the prevalence and burden of OM were 3.5% and 497.35 million US dollars, respectively. Patients who were 0–9 years old accounted for 59.7% of the cases and 55.2% of the total cost. Among adults, the total and perpatient costs were highest among 50–59-year-old adults. Direct medical costs and outpatient costs accounted for large proportions of the total cost (86.3% and 88.3%, resp.).* Conclusion*. The economic burden of OM decreased after the introduction of the pneumococcal conjugate vaccines. However, OM is still associated with a relatively large burden, especially among adults, and interventions are needed to reduce the burden of OM in this population.

## 1. Introduction

Otitis media (OM) is a common middle-ear condition and is particularly common among children [1, 2]. OM typically presents as acute OM (AOM), which is acute inflammation of Eustachian tube because of viral and bacterial infections, or as OM with effusion (OME), which is accumulation of fluid in the middle ear without acute symptoms, such as pain and fever [1–4]. Several studies have revealed that >80% of children experience AOM before they are 5 years old and that approximately 90% of children experience OME before they reach school age, especially 6-month-olds to 4-year-olds [5, 6]. Furthermore, a Korean study revealed that children who were <9 years old accounted for >90% of cases that required myringotomy or v-tube to treat AOM [4]. Other studies have found that recurrent AOM during early childhood is associated with Eustachian tube inefficiency and that adults with a history of childhood OM exhibit poor tube function [2, 7]. Moreover, chronic suppurative OM is the most common cause of hearing disabilities in developing countries [8], which indicates that childhood OM may exhibit a complicated course and sequelae with long-term effects. The annual economic burden of OM in the US is approximately 3–5 billion US dollars (USD), although the real cost may be higher, because of underestimated indirect costs [9]. The total cost of AOM in Korea was reported to be approximately 530.11 million USD in 2004 [4].

Approximately 70% of all AOM cases are caused by bacteria [3]. The three major bacterial pathogens are* Streptococcus pneumoniae*,* Haemophilus influenzae*, and* Moraxella catarrhalis*, with* S. pneumoniae* being the most frequent pathogen in children with AOM; treatment failure is often related to antibiotic resistance [10–13]. The incidence of OM among <10-year-old children was reduced by 22% after the introduction of the 7-valent pneumococcal conjugate vaccine (PCV-7), and a further 19% reduction was observed after the introduction of PCV-13 in the UK [14]. However, in Korea, only one report has described the economic burden of OM using National Health Insurance Service (NHIS) claims data from 2003, which was before the Korean introduction of the PCVs [4]. Thus, it is unclear whether the PCVs have affected the prevalence and burden of OM in Korea. And the economic burden of OM on each age group is unclear. Therefore, this study aimed to estimate the prevalence and economic burden of OM after the PCVs were implemented in the Korea.

## 2. Methods

### 2.1. Ethical Statement

This study was approved by the institutional review board (1040548-KU-IRB-13-164-A-1).

### 2.2. Estimation of OM Cases and Cost of Illness Analysis

The Korean healthcare system is supported by a single national administrative body (the NHIS). Approximately 97.3% of Koreans were covered by the NHIS in June 2015, and >99% of Koreans are covered by some form of health care coverage (including Medical Aid) [15]. Once an NHIS beneficiary receives healthcare in a medical institution, any related records are accumulated in a database that is managed by the NHIS. Therefore, NHIS claims data provide medical information that is representative of the entire Korean population. This study used NHIS claims data from January 1 to December 31, 2012, to estimate the Korean prevalence and burden of OM.

Cases of OM were calculated using a prevalence-based approach and were defined as claims of at least 1 hospitalization or at least 2 outpatient visits during the study period. The diagnostic codes were based on the World Health Organization's tenth version of the International Classification of Disease codes: H65 (nonsuppurative OM), H66 (suppurative and unspecified OM), H67 (OM in diseases classified elsewhere), H68 (Eustachian salpingitis and obstruction), and H70 (mastoiditis and related conditions) [16]. To calculate the prevalence of OM, we divided the number of patients with OM by the registered population of Korean residents from 2012 [17].

We analyzed the economic burden of OM from the societal perspective, which refers to a comprehensive concept that covers medical costs (e.g., insured and uninsured medical costs), nonmedical costs (e.g., transportation and caregiver costs), and lost productivity that is related to work loss or premature death [18]. Direct medical costs were estimated using the macrocosting method, and indirect costs were estimated using the human capital approach. All costs were converted from Korean won (KRW) to US dollars (USD) using the mean exchange rate from 2012 (1 USD = 1,126.76 KRW) [19].

### 2.3. Estimation of Direct Costs

We used NHIS claims data to directly calculate insured costs, such as outpatient and inpatient medical costs. Uncovered medical costs were estimated by multiplying the uninsured cost rate (uncovered medical costs divided by total medical costs) from perpatient analyses that were performed by the NHIS [20]. Pharmaceuticals costs were estimated using outpatients pharmacy costs, as a proportion of all medical costs, from the 2012 National Health Insurance Statistical Annual Report (Table 1) [21].

Direct medical costs were estimated using the following formula:

(1) Medical  cost=∑ij∑InNHIij+OPij+OutNHIij+OPij+OutDij,

where *i* indicates age (0,1, 2,…, *n* years), *j* indicates sex (female, male), InNHI indicates the NHI payment for hospitalization, OutNHI indicates the NHI payment for outpatient care, OP indicates the uninsured medical costs (out of pocket payments), and OutD indicates outpatient drug costs.

Nonmedical costs were divided into a transportation cost and a caregiver cost. For the transportation cost, the numbers of outpatient visits and hospitalizations were derived from the NHIS claims data and were multiplied by the pervisit transportation cost from the Korea Health Panel data [22]. For the caregiver cost, the number of outpatient and hospitalization days was derived from the NHIS claims data and was multiplied by the average daily cost for paid caregivers, based on the Korea Patient Helper Society suggestions and the rate of caregiver utilization from the Korea Health Panel data (Table 1) [22, 23]. However, we assumed that 20–69-year-old patients had no caregiver costs during outpatient visits and that the caregiver costs for outpatient visits were 1/3 of the daily caregiver cost (55.861 USD). The transportation costs were defined as 2.169 USD per outpatient visit and 20.246 USD per hospitalization.

Nonmedical costs were calculated using the following formula:

(2) Non-medical cost=∑ij∑InVij×InTij+OutVij×OutTij+InDij×CG+OutVij×CG×13−∑j∑i=2069OutVij×CG×13,

where *i* indicates age (0,1, 2,…, *n* years), *j* indicates sex (female, male), InV indicates the number of inpatient visits, InT indicates the round-trip transportation cost per inpatient visit, OutV indicates the number of outpatient visits, OutT indicates the round-trip transportation cost per outpatient visit, InD indicates the length of hospitalization, and CG indicates the daily caregiver cost.

### 2.4. Estimation of Indirect Costs

Indirect costs were defined as work-loss costs (interruption of work to visit medical institutions) and future income loss because of premature death. The numbers of outpatient visits and hospitalization days were calculated from the NHIS claims data and were multiplied by the daily average wage for various types of employment, based on the Ministry of Employment and Labor's 2012 survey report (Table 1) [24]. However, we assumed that only 20–69-year-old patients would experience work loss, and the amount of loss because of outpatient visits was defined as 1/3 of the daily average wage. The average daily wages for the analyses were 60.235 USD for patients who were 20–29 years old, 95.301 USD for patients who were 30–39 years old, 120.246 USD for patients who were 40–49 years old, 110.365 USD for patients who were 50–59 years old, and 69.841 USD for patients who were 60–69 years old.

Work-loss costs were estimated using the following formula:

(3) Work-loss cost=∑ij∑InDij×ICij+OutVij×13×ICij,

where *i* indicates age (0,1, 2,…, *n* years), *j* indicates sex (female, male), InD indicates the number of hospitalization days, IC indicates the daily average wage, and OutV indicates the number of outpatient visits.

To estimate future income loss, we calculated the rates of OM-related death from Statistics Korea. The rates were multiplied by the age- and sex-specific loss amounts that were calculated using the average annual nominal wage from the year after death to the average life expectancy and a discount rate (Table 1) [25]. However, we assumed that individuals who were ≥70 years old would not earn future income. The cost of lost future income was estimated using the following formula:

(4) $$\begin{array}{c} \text{Permature death cost}=\underset{i}{\sum }\underset{j}{\sum }\overset{n}{\underset{k}{\sum }}\left(\;N_{ij}\times \frac{Y_{ij\left(t+k\right)}}{{\left(1+r\right)}^{k}}\;\right), \end{array}$$

where *i* indicates age (0,1, 2,…, *n* years), *j* indicates sex (female, male), *N* indicates the number of deaths (*k*:  1,2,…, *n* [*n* indicates the difference between life expectancy and death]), *Y*_*ij*(*t* + *k*)_ indicates the average annual income at the age of *t* + *k*, and *r* indicates the discount rate.

## 3. Results

In 2012, 1,788,303 Korean patients visited medical institutions for treatment of OM (at least one hospitalization or at least two outpatient visits). Patients who were 0–9 years old accounted for 59.7% of all cases. The total prevalence of OM was 3.5%, although the prevalence was 22.9% among 0–9-year-old patients (Table 2). The mean annual number of outpatient visits per patient was 6.47, and the highest number was 7.38 for 0–9-year-old patients. The mean number of hospitalizations per 100 patients was 2.29, and the highest number was 4.81 for 50–59-year-old patients. In contrast, 0–9-year-old patients exhibited a rate of 2 hospitalizations per 100 patients. In addition, the mean duration per hospitalization was 5.19 days, which tended to increase with age (0–9-year old patients: 4.67 days, ≥80-year-old patients: 10.96 days) (Table 3). The numbers of outpatient visits per patient were 6.75 for male patients and 6.22 for female patients, the numbers of hospitalizations per 100 patients were 2.33 for male patients and 2.25 for female patients, and the mean durations per hospitalization were 5.06 days for male patients and 5.32 days for female patients; there were no significant sex-specific differences (Table 4).

Table 5 shows the economic burden of OM among Koreans based on the direct and indirect costs in 2012. The total burden was 497.35 million USD, which accounted for approximately 0.04% of the Korean gross domestic product in 2012. Direct costs were 429.04 million USD (86.3%), and indirect costs were 68.31 million USD (13.7%). Among the direct costs, medical costs were 279.81 million USD (65.2%) and nonmedical costs were 149.22 million USD (34.8%). Among the various specific medical costs, outpatient costs were 135.55 million USD (31.6% of total direct costs), which were followed by uninsured costs (14.7%), pharmaceutical costs (11.4%), and hospitalization costs (7.5%). Nonmedical costs included caregiver costs of 123.29 million USD (28.7% of total direct costs) and transportation costs of 25.93 million USD (6.0%).

Indirect costs were calculated as the sum of work-loss costs and lost future income because of OM-related death. However, we only identified 2 OM-related deaths (both women who were in their 80s); thus, we defined the cost due to premature mortality as 0 USD. The work-loss costs were 68.31 million USD.

The economic burdens of OM according to age group and inpatient/outpatient care are presented in Table 6. The economic burden of inpatient care was 58.12 million USD (11.7% of the total cost), compared to 439.24 million USD (88.3%) for outpatient care. The direct costs of hospitalization were 51.13 million USD (88.0%), and the indirect costs were 7.00 million USD (12.0%). In contrast, the direct costs of outpatient care were 377.93 million USD (86.0%), and the indirect costs were 61.31 million USD (14.0%). Thus, indirect costs were 2% higher for outpatients, compared to inpatients.

The economic burden of OM-related hospitalization among 0–9-year-old patients was 16.76 million USD (6.1% of the total cost in that age group) and the cost of outpatient care was 257.87 million USD (93.9%). Among 50–59-year-old patients, the economic burden of hospitalization was 13.49 million USD (24.9% of the total cost in that age group) and the cost of outpatient care was 40.72 million USD (75.1%). The highest proportion of inpatient economic burden was observed among 0–9-year-old patients (28.8%), which was followed by 23.2% for 50–59-year-old patients and 15.9% for 40–49-year-old patients. The highest proportion of outpatient economic burden was observed among 0–9-year-old patients (58.7%), which was followed by 10.9% for 50–59-year-old patients and 8.4% for 40–49-year-old patients. The perpatient economic burden was 278.11 USD. The highest perpatient burden was observed among 50–59-year-old patients (438.76 USD), which was followed by 393.79 USD for 60–69-year-old patients, 382.52 USD for 40–49-year-old patients, and 312.65 USD for 70–79-year-old patients.

## 4. Discussion

The present study evaluated the prevalence and economic burden of OM in Korea during 2012. The prevalence of OM was 3.5%, with 6.47 annual outpatient visits per patient. Patients who were 0–9 years old had the highest medical usage, with a prevalence of 22.9% and 7.38 annual outpatient visits. However, hospitalizations were most common among 50–59-year-old patients (4.81 per year), and 0–9-year-old patients exhibited a relatively low hospitalization rate (2.00 per year). The total economic burden of OM was estimated to be 497.35 million USD, which mainly included direct medical costs (outpatient, caregiver, and uninsured medical costs, resp.). The indirect costs mainly included work-loss costs, as mortality-related costs were minimal. Patients who were 0–9 years old accounted for 28.3% of the total costs and were followed by the 50–59-year-old and 40–49-year-old groups. However, the perpatient costs were highest in the 50–59-year-old group, which was followed by the 60–69-year-old and 40–49-year-old groups. Studies have focused on the economic costs and the prevalence of OM in children; however, few studies have focused on different age groups as in our study [4, 14, 26, 27]. As far as we know, this is the first study in Korea to analyze the economic costs and the hospitalizations for OM in each age group. The reported prevalence of OM varies according to countries and ethnic groups [28]. This is complicated by the fact that there is considerable variation, among studies, in the definition of the disease. For example, one study stated that the prevalence of chronic OM ranged from 1% to 46%, which is clearly a wide range. The Inuit represent the ethnic group with the highest prevalence of OM, with prevalence ranging from 12 to 46%. The lowest prevalence of OM was under 1% and reported in the United States and England. A further study estimated that the prevalence of AOM and chronic suppurative OM were 0.22% and 2.89%, respectively, among high-income residents of the Asia-Pacific region [26]. Our study reported the prevalence of OM in South Korea as 3.5% and this finding is consistent with previous studies.

While there is wide variation in the prevalence of OM among different populations, the prevalence of OM is declining worldwide with improvements in the use of antibiotics and vaccinations, healthcare accessibility, and health behaviors [29]. Nevertheless, OM remains a leading global cause of healthcare visits and drug prescriptions [26] and can negatively affect quality of life, as it can cause permanent hearing impairment. For example, OM accounted for 1,806,500 disability-adjusted life years (DALYs) globally during 2013, which corresponds to a 3.1% increase (versus 2005) and is in stark contrast with an 18.5% decrease in the burden of communicable diseases and a 2.5% decrease in the burden of all diseases [30]. The OM-related DALYs in Korea decreased from 19.86 per 100,000 in 1990 to 17.02 per 100,000 in 2013. However, the decreasing trend for OM's DALY in Korea is relatively small, compared to other communicable diseases (decreased from 2,365 DALYs per 100,000 in 1990 to 1,460 DALYs per 100,000 in 2013). Thus, the burden of communicable disease decreased by 40% over 23 years, and the burden of all disease also decreased from 25,435 DALYs per 100,000 in 1990 to 22,925 per 100,000 in 2013 [31], which indicates the increasing relative importance of OM among communicable and all diseases. For example, one study found that OM ranked 13th among men and 4th among women in terms of DALY, with OM-related DALYs among women increasing from 384 per 100,000 in 2000 to 493 per 100,000 in 2010 [32]. In addition, we found that 3.5% of the Korean population used healthcare because of OM, and approximately 20% of the 0–9-year-old age group experienced OM. Furthermore, the economic burden of OM was estimated to be 497.35 million USD (approximately 0.04% of the Korean gross domestic product in 2012), which is higher than the burdens for allergic rhinitis [33] or hepatitis A and hepatitis C [34]. Moreover, the economic burden of OM accounted for more than half of the burden of breast cancer [35]. The economic burden of OM is substantial, despite the fact that there is considerable variability among studies in terms of the definitions and the inclusion criteria used as the basis for the calculation of the economic impact. For example, one study in the US estimated the economic burden of OM in children at 2.88 billion USD in 2008 [36]. Another study in Australia estimated the economic burden of OM in a cohort of children from birth to the age of four years as 138~339 million USD in 2008 [37]. These results show that OM represents a significant public health burden, even in developed countries, including Korea. Furthermore, the need for strategies to minimize this burden should be highlighted, as communicable diseases generally affect the most disadvantaged sections of the population, even in developed countries [38].

Interestingly, we found that the adult prevalence of OM was highest among the 70–79-year-old age group and lowest among the 20–29-year-old age group. Another study estimated the prevalence of chronic OM among adults based on otoendoscopy findings [29] and revealed that the age-specific prevalence ranged from 1.1% to 8.5%, with the highest prevalence observed among adults who were >70 years old. Therefore, the high prevalence in the 70–79-year-old age group is likely mainly related to chronic OM. Among children and adolescents, the highest prevalence was observed in the 0–9-year-old age group (59.7% of all cases), and 0–19-year-old patients accounted for 67.2% of all cases. Other studies have also reported relatively high prevalence among children and adolescents [4, 26], with a 0–16-year-old age group accounting for 76.4% of all outpatient visits and 75.9% of the total economic burden in Korea during 2004 [4].

To minimize the burden of OM, PCVs were developed for* S. pneumoniae*. In the UK, the incidence of OM among <10-year-old children was significantly reduced after the implementation of the PCV-7 and PCV-13 vaccines. In addition, the introduction of the PCV-13 vaccine in 2010 reduced the American incidence of OM-related visits, especially among patients who were <2 years old [39]. In Korea, the PCV-7 and PCV-13 vaccines were introduced in 2003 and 2010, respectively, and the PCV-13 vaccine has been commonly used as part of the National immunization program (NIP) since 2014. The economic burden of OM in 2004 was estimated to be 530.11 million USD, while the burden in the present study was estimated to be 497.35 million USD. Therefore, the introduction of the PCV-7 and PCV-13 vaccines appears to have provided some reduction in the economic burden. In fact, a comparison of the 1992–1998 cohort (before pneumococcal vaccination) and the 2000–2003 cohort (after implementing the vaccination) reveals that the proportion of* S. pneumoniae* decreased from 48% to 31% and that nontypable* H. influenzae* has become the most prevalent organism among children with severe or refractory AOM.

Many studies regarding OM have focused on children and adolescents [29], although the burden of OM among adults is also becoming important, based on the broader use of the PCV vaccines (which can prevent OM especially in the children). For example, adult cases accounted for 36.3% of outpatient costs and 67.8% of inpatient costs, and the highest perpatient costs were observed in the 50–59-year-old group, rather than among children or adolescents. Furthermore, the highest severity (measured based on admissions per patient) was observed in the 50–59-year-old group. These results highlight the fact that the adult burden of OM requires further attention and interventions to manage this burden. For example, monitoring the pathogens that are responsible for OM can improve antimicrobial therapy and prevent important sequelae, such as hearing loss.

This study has several limitations that warrant consideration. First, we used NHIS claims data to estimate the economic burden of OM, and this burden may be underestimated if a significant proportion of patients do not seek medical treatment. Moreover, the economic burden could increase even further if the cost of school absenteeism is taken into account. Second, the administrative nature of claims data does not guarantee the accuracy of an OM diagnosis, although these characteristics have a limited effect in Korea [40]. Third, the cross-sectional design makes it difficult to accurately estimate the effect of vaccination on the prevalence and burden of OM. Fourth, we did not consider the otopathogen, which makes it difficult to evaluate the effects of the PCV-7 and PCV-13 vaccines, despite the decrease in the economic burden. Therefore, additional research is needed to evaluate the effects of including PCV-13 in the NIP. Fifth, daycare attendance is a significant risk factor for AOM [25], and the Korean government recently increased its funding of daycare centers, with approximately 77.3% of <5-year-old children attending a daycare center [29]. Therefore, further research is needed to evaluate how promoting daycare may affect the prevalence and burden of OM.

In conclusion, the present study used nationally representative data to estimate the prevalence and economic burden of OM in Korea during 2012. Our results indicate that the prevalence of OM was 3.5% and the estimated economic burden of OM was 497.35 million USD, which is lower than the reported economic burden from 2004. These decreases may be related to the use of the PCVs. Nevertheless, the Korean burden of OM remains substantial, and these trends are consistent with worldwide trends. Furthermore, OM is becoming increasingly important among adults (versus children and adolescents), with the 50–59-year-old group exhibiting the highest perpatient cost and the second highest total cost. Therefore, policy interventions are needed to reduce the adult burden of OM, and further research is needed to evaluate the effects of the PCVs on the prevalence and economic burden of OM.

## Figures and Tables

**Table 1:** Classification and data sources for costs.

Classification	Services included in category	Data source
*Direct cost*		
Direct medical costs	Outpatient medical cost	NHIS claims data between 2012.1.1 and 2012.12.31.
	Inpatient medical cost	NHIS claims data between 2012.1.1 and 2012.12.31.
	Uninsured medical cost (out of pocket cost)	Using a ratio for noninsured medical cost per total medical cost surveyed at NHIS
	Pharmaceuticals cost	Using a ratio for pharmaceuticals cost per total medical cost published at NHIS and HIRA 2012 National Health Insurance Statistical Yearbook
Direct nonmedical costs	Transportation cost	Korea Health Panel Data surveyed at NHIS and KIHASA
	Caregiver's cost	(1) Using a ratio of paid caregiver of Korea Health Panel data surveyed at NHIS and KIHASA (2) Using a cost for paid caregiver's average cost announced at The Korean Patient Helper Society
*Indirect cost*		
Productivity loss	Due to work loss	2012 Survey Report on Labor Conditions by Employment Type surveyed at Ministry of Employment and Labor
	Due to premature death	Statistics Korea Data in 2012

^*∗*^NHIS: national health insurance service; KIHASA: Korea institute for health and social affairs; HIRA: health insurance review and assessment service.

**Table 2:** Prevalence rate of otitis media by age and gender in 2012.

Age	Male			Female			Total		
	Number of patients	%	Prevalence	Number of patients	%	Prevalence	Number of patients	%	Prevalence
0–9	559,308	64.9	23.2	508,340	54.9	22.5	1,067,648	59.7	22.9
10–19	79,274	9.2	2.3	67,147	7.3	2.2	146,421	8.2	2.3
20–29	20,951	2.4	0.6	37,511	4.1	1.2	58,462	3.3	0.9
30–39	37,085	4.3	0.9	66,041	7.1	1.6	103,126	5.8	1.3
40–49	45,872	5.3	1.0	63,432	6.9	1.5	109,304	6.1	1.2
50–59	48,841	5.7	1.2	74,714	8.1	1.9	123,555	6.9	1.6
60–69	37,855	4.4	1.8	53,940	5.8	2.4	91,795	5.1	2.1
70–79	26,568	3.1	2.1	41,977	4.5	2.4	68,545	3.8	2.3
80+	6,036	0.7	1.9	13,411	1.5	1.7	19,447	1.1	1.8

Total	861,790	100.0	3.4	926,513	100.0	3.6	1,788,303	100.0	3.5

**Table 3:** Rates of inpatient admissions and outpatient visits due to otitis media by age.

Age	Outpatient		Inpatient		
	Number of outpatient visits	Number of outpatient visits (per 1 OM^*∗*^ patient)	Number of inpatient admissions	Number of inpatient admissions (per 100 OM^*∗*^ patient)	Inpatient hospital days (per 1 OM^*∗*^ inpatient admission)
0–9	7,877,576	7.38	21,384	2.00	4.67
10–19	596,280	4.07	1,441	0.98	4.86
20–29	217,858	3.73	944	1.64	5.54
30–39	428,285	4.15	1,924	1.87	5.47
40–49	550,212	5.03	4,086	3.74	5.67
50–59	710,480	5.75	5,937	4.81	5.82
60–69	585,154	6.37	3,694	4.02	5.93
70–79	481,091	7.02	1,276	1.86	6.16
80+	131,155	6.74	211	1.09	10.96

Total	11,578,091	6.47	40,897	2.29	5.19

^*∗*^OM: otitis media.

**Table 4:** Rates of inpatient admissions and outpatient visits due to otitis media by gender.

Gender	Outpatient		Inpatient		
	Number of outpatient visits	Number of outpatient visits (per 1 OM^*∗*^ patient)	Number of inpatient admissions	Number of inpatient admissions (per 100 OM^*∗*^ patient)	Days of inpatient admission (per 1 OM^*∗*^ inpatient admission)
Male	5,815,495	6.75	20,093	2.33	5.06
Female	5,762,596	6.22	20,804	2.25	5.32

Total	11,578,091	6.47	40,897	2.29	5.19

^*∗*^OM: otitis media.

**Table 5:** Economic burden of disease due to otitis media for Koreans in 2012.

Classification		Cost^*∗*^
*Direct costs*		
Direct medical costs	Outpatient medical cost	135.55
	Inpatient medical cost	32.06
	Uninsured medical cost (out of pocket cost)	63.25
	Pharmaceuticals cost	48.95
Direct nonmedical costs	Transportation cost for hospital visits	25.93
	Caregiver's cost	123.29
Total, direct costs		429.04
*Indirect costs*		
Productivity loss		68.31
Total, indirect costs		68.31

Total costs		497.35

^*∗*^Unit: 1 million US dollar.

1 US dollar = 1,126.76 Korean won (mean exchange rate of 2012).

**Table 6:** Economic burden of disease due to otitis media by age group in 2012.

Age	Inpatient				Outpatient				Total				
	Number of outpatient visits	Direct cost^*∗*^	Indirect cost^*∗*^	Total cost^*∗*^	Total days of inpatient admission	Direct cost^*∗*^	Indirect cost^*∗*^	Total cost^*∗*^	Number of patients	Direct cost^*∗*^	Indirect cost^*∗*^	Total cost^*∗*^	Per capita
0–9	7,877,576	16.76	—	16.76	99,773	257.87	—	257.87	1,067,648	274.62	—	274.62	257.22
10–19	596,280	1.90	—	1.90	7,004	21.74	—	21.74	146,421	23.64	—	23.64	161.44
20–29	217,858	1.57	0.31	1.88	5,228	6.20	4.27	10.47	58,462	7.77	4.58	12.35	211.22
30–39	428,285	3.18	0.89	4.07	10,532	11.66	11.90	23.56	103,126	14.84	12.79	27.63	267.91
40–49	550,212	7.24	2.01	9.25	23,158	16.33	16.23	32.56	109,304	23.57	18.24	41.81	382.52
50–59	710,480	10.88	2.62	13.49	34,540	22.31	18.41	40.72	123,555	33.18	21.03	54.21	438.76
60–69	585,154	6.82	1.17	7.99	21,856	17.66	10.50	28.16	91,795	24.48	11.67	36.15	393.79
70–79	481,091	2.37	—	2.37	7,864	19.06	—	19.06	68,545	21.43	—	21.43	312.65
80+	131,155	0.41	—	0.41	2,312	5.10	—	5.10	19,447	5.51	—	5.51	283.26

Total	11,578,091	51.13	7.00	58.12	40,897	377.93	61.31	439.24	1,788,303	429.04	48.31	497.35	278.11

^*∗*^Unit: 1 million US dollar.

1 US dollar = 1,126.76 Korean won (mean exchange rate of 2012).
